# The impact of artificial intelligence on biomarker discovery

**DOI:** 10.1042/ETLS20243003

**Published:** 2025-09-09

**Authors:** Hira Javaid, Constantin Cezar Petrescu, Lisa J. Schmunk, Jack M. Monahan, Paul O'Reilly, Manik Garg, Leona McGirr, Mahmoud T. Khasawneh, Mustafa Al Lail, Deepak Ganta, Thomas M. Stubbs, Benjamin B. Sun, Dimitrios Vitsios, Daniel E. Martin-Herranz

**Affiliations:** 1 Hurdle.bio / Chronomics Ltd., London, UK; 2 Biomodal Ltd, The Trinity Building, Chesterford Business Park, Cambridge, UK; 3 Sonrai Analytics Ltd, Queens Road, Belfast, Northern Ireland, UK; 4 Centre for Genomics Research, Discovery Sciences, BioPharmaceuticals R&D, AstraZeneca, Cambridge, UK; 5 Texas A&M International University, Laredo, Texas, U.S.A.; 6 Bristol Myers Squibb, New York, U.S.A.

**Keywords:** biomarkers, diagnostics, biomarker discovery, artificial intelligence, multi-omics, electronic health records, multi-modal data

## Abstract

Artificial intelligence (AI) is transforming many fields, including healthcare and medicine. In biomarker discovery, AI algorithms have had a profound impact, thanks to their ability to derive insights from complex high-dimensional datasets and integrate multi-modal datatypes (such as omics, electronic health records, imaging or sensor and wearable data). However, despite the proliferation of AI-powered biomarkers, significant hurdles still remain in translating them to the clinic and driving adoption, including lack of population diversity, difficulties accessing harmonised data, costly and time-consuming clinical studies, evolving AI regulatory frameworks and absence of scalable diagnostic infrastructure. Here, we provide an overview of the AI toolkit available for biomarker discovery, and we discuss exciting examples of AI-powered biomarkers across therapeutic areas. Finally, we address the challenges ahead of us to ensure that these technologies reach patients and users globally and unlock a new era of fast innovation for precision medicine.

## Introduction

Artificial intelligence (AI) can be broadly defined as ‘any technique that enables computers to mimic human behaviour and reproduce or excel over human decision-making to solve complex tasks independently or with minimal human intervention’ [1]. AI has revolutionised many domains that had traditionally been difficult to tackle computationally. More recently, transformer-based large language models (LLMs) have made newspaper headlines with their abilities to generalise across many natural language and computer vision tasks [2,3]. Consequently, the power of LLMs has triggered an AI race across technology companies.

AI-powered solutions are paving the way towards a significant impact of AI in healthcare. Some examples include protein structure prediction for drug discovery [4], applications in personalised medicine [5] and medicine-specific foundational models [6] (which can help to automate and increase efficiency in clinical workflows). With the rapid increase in large multi-omics and multi-modal healthcare datasets and deeply phenotyped biobanks, AI algorithms have become a cornerstone of engaging with big data to discover new biomarkers [7–9]. This review uses a broad definition of AI, including both classical machine learning (ML) and more recent deep learning (DL) algorithms. We discuss how researchers have used AI in the context of biomarker discovery in humans and provide an industry view on the areas where it has had more impact, while discussing some of the challenges to translate these biomarkers into everyday clinical practices.

## Biomarker nomenclature and classification

A biomarker is defined as ‘a characteristic that is measured as an indicator of normal biological processes, pathogenic processes or responses to an exposure or intervention, including therapeutic interventions’ [10,11]. The FDA-NIH Biomarkers, EndpointS and other Tools (BEST) biomarker classification defines seven categories of biomarkers [10], which we have summarised below [12]:

1. susceptibility/risk biomarkers, which indicate a person’s risk for developing a specific disease;
2. diagnostic biomarkers, which detect the presence of a disease or disease subtype;
3. monitoring biomarkers, which assess the status of a disease;
4. prognostic biomarkers, which give the likelihood of a clinical event;
5. predictive biomarkers, which identify individuals more likely to have a favourable response to treatment;
6. pharmacodynamic/response biomarkers, which show that a response has occurred due to a treatment; and
7. safety biomarkers, which indicate the likelihood of or the presence of toxicity due to a medical product.

**Figure 1:**
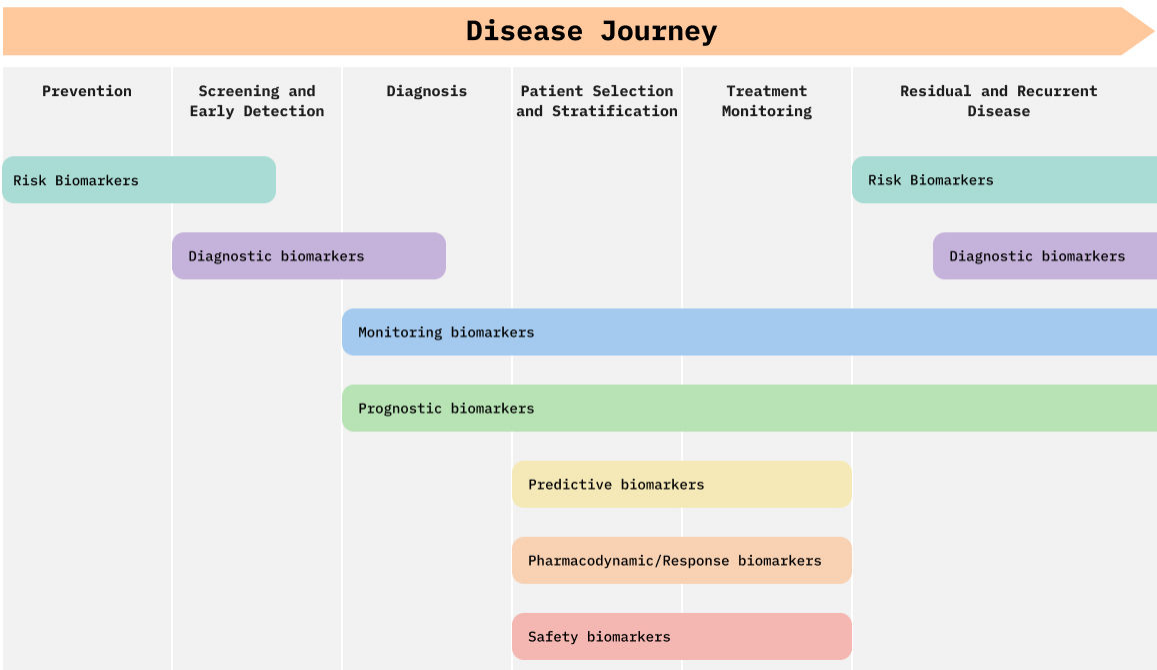
Biomarker categories along the disease journey. The seven biomarker categories come into play at different stages of the patient disease journey, from prevention to diagnosis, treatment and recurrence.

These biomarker categories may be useful at different stages of the disease journey (Figure 1). Furthermore, companion diagnostics are medical devices that are essential for the safe and effective use of a corresponding drug [13]. This includes predictive biomarkers of drug response (used to stratify patients into responders and non-responders or to identify individuals at increased risk of side effects) and safety biomarkers (to monitor a safe response to treatment and adjust dose accordingly) [13].

## The toolbox for AI-powered biomarker discovery

### Datatypes used in biomarker discovery

Traditional biomarkers can be expressed as a single number of a single datatype, such as HbA1c (glycated haemoglobin) or cholesterol. AI algorithms become useful in the presence of highly dimensional datasets, which have surged in the last decade [14]. There are distinct types of biological data, which can include different modalities:

- Omics data: Molecular data such as genomics, epigenomics, transcriptomics, proteomics, glycomics, metabolomics or microbiomics. When more than one of these omics datatypes is leveraged, this is generally referred to as multi-omics [15]. This has gained particular attention in the context of single-cell approaches, where multiple omics layers are profiled at the same time from a single cell [16,17].
- Electronic health records (EHRs) and questionnaire-based data: They can contain structured data (such as lab test results, demographic and lifestyle/environmental data) or unstructured data (such as text from clinical notes).
- Imaging data: Such as radiology data [like computed tomography (CT) scans], histological images (digital pathology), electrocardiograms (ECG), electroencephalography (EEG), magnetic resonance imaging (MRI) or positron emission tomography (PET) scans.
- Sensor data: Such as that derived from wearable devices.

AI algorithms can be used for batch correction, data harmonisation and dimensionality reduction within one datatype (e.g. when dealing with high-dimensional single-cell transcriptomic data) [18]; to integrate different datatypes of the same modality (e.g. multi-omics approaches that map proteomics signatures to epigenomic data) [19]; or to combine different data modalities, which is referred to as multi-modal (e.g. combining genetics and imaging data to discover new disease subtypes) [20].

### Multi-modal data sources

Biobanks now hold vast amounts of multi-modal data that can be accessed for biomarker discovery (Table 1). Disease-agnostic biobanks are set up with the aim of surveying the health of a population over time. They often follow a prospective cohort design and recruit participants to perform a series of baseline tests and store tissue and data for future research use. This cross-sectional data can be enhanced by regular follow-ups with questionnaires, imaging and re-sampling, as well as linking to national healthcare records. While especially omics data generation has depended on collaborations between pharmaceutical industry, governments and private research funders (e.g. genomics and proteomics data in the UK Biobank [34]), the data are now available for general use.

**Table 1:** Examples of global multi-modal datasets that can be leveraged for AI-powered biomarker discovery.

Region	Country	Biobank name	Type	Number of participants	Recruitment years	Age at recruitment	Data modalities	Access	Longitudinal sampling	Number of years of follow-up	References
Africa	Multiple African nations	H3Africa biorepositories	Biorepository	~100,000	2012–Present	NA	Omics	Application-based	No	NA	[21]
Asia	China	China Kadoorie Biobank	Prospective	~512,000	2004–2008	30–79 years	Omics, EHRs	Application-based; genetic data restricted	Surveys only	~15 years	[22]
	Japan	Tohoku Medical Megabank	Prospective (earthquake survivor regions)	~150,000	2013–2018	~3/4 20+ years, ~1/4 < 20 years	Omics, EHRs	Application-based for academia & industry	Yes, every 5 years	~10+ years	[23]
	Japan	BioBank Japan	Disease-focused	~270,000	2003–2008 & 2012–2017	Median age: ~62 years (2003–2008)	Omics, EHRs	Application-based for academia & industry	No	~10+ years	[24]
Europe	United Kingdom	UK Biobank	Prospective	~500,000	2006–2010	40–69 years	Omics, EHRs and imaging	Application-based for academia & industry	Yes	~15+ years	[25]
	Estonia	Estonian Biobank	Prospective	~200,000	2002–2015 & 2018–2024	18+ years	Omics, EHRs	Application-based for academia & industry	Surveys only	Up to 20+ years	[26]
	Finland	FinnGen	Disease-focused (2/5) + Prospective (3/5)	~500,000	2017–Present (legacy before 2017)	Median age: ~63 years	Omics, EHRs	Application-based for academia & industry	No	~7+ years	[27]
Middle East	Qatar	Qatar Biobank	Prospective	~45,000 (target 60,000)	2012–Present	18+ years	Omics, EHRs and imaging	Application-based for medical researchers	Yes	~10+ years	[28]
North America	USA	All of Us	Prospective	~1,000,000	2018–Present	18+ years	Omics, EHRs, sensor data	Restricted; application-based for academia	Yes	~5+ years	[29]
	Canada	CanPath	Prospective	~330,000	2008–Present	30–74 years	Omics, EHRs	Application-based for academia & industry	Yes	~12+ years	[30]
	USA	Million Veteran Program	Prospective	~1,000,000	2011–Present	Median age: ~66 years ( >90% male)	Omics, EHRs	Restricted; Application-based for academia	No	~10+ years	[31]
South America	Brazil	Biobank Barretos Cancer Hospital	Disease-focused (cancer)	~50,000	2006–Present	NA	Omics, EHRs	NA	NA	NA	[32]
	Mexico	Mexico City prospective study	Prospective	~110,000	1998–2004	NA	Omics, EHRs (mortality records)	Application-based	Re-survey of ~10,000 in 2015 and 2019	~20+ years	[33]

Beyond disease-agnostic national or private biobanks, data valuable for biomarker discovery can be found in disease-focused biobanks and cohort studies, commercial research datasets (e.g. clinical trials), as well as real-world data (e.g. from EHRs, insurance records and consumer testing companies). Longitudinal datasets with repeat sampling of multi-modal datatypes provide opportunities for direct assessment of health progression [35]. Family studies (e.g. trios, parent–child and multigenerational) have not been widely used in biomarker discovery yet [36]. Biases introduced by minimal phenotyping and the self-reporting of certain phenotypes and pathologies may lower specificity for biomarker discovery [37].

Given the large-scale missingness, especially in omics and EHR data, imputation and synthetic datasets can be beneficial for biomarker discovery. Imputation of missing values across all datatypes is non-trivial, with approaches for omics and EHR data previously reviewed [38,39]. For example, genetic information can be leveraged to improve imputation of missing traits in EHRs [40], while DL-based methods can enable biomarker feature imputation [41]. Recently, generative AI approaches [such as generative adversarial networks (GANs) or transformers] have been leveraged to create synthetic datasets across EHRs (e.g. EHRSafe) [42] or epigenetic data (e.g. MethylGPT) [43].

Access to individual level data may be open access or restricted (see Table 1) and is increasingly provided in Trusted Research Environments (TREs). While this facilitates dataset maintenance and within-dataset harmonisation, it precludes training across multiple datasets and biobanks held in different systems. Federated learning approaches may be considered in these cases if data cannot be combined locally [44,45].

The data required to train an AI biomarker model will depend on the biomarker type (Figure 1). Most biomarker discovery models to date have used supervised learning, where the outcome variable needs to be defined. For example, in order to develop a susceptibility/risk biomarker, data with disease outcomes in a large population are essential (e.g. time-to-disease models), which needs prospective studies with significant follow-up data. For predictive and pharmacodynamic/response biomarkers, medication records will be needed. Sometimes the outcome variable is not clearly defined, such as in the case of new phenotypes (e.g. chronic inflammation, a hallmark of ageing) or when dealing with different disease billing codes. Importantly, biomarkers can themselves help to identify disease subtypes and fine-tune disease classification, which can lead to improvements in biomarker discovery [46].

A major consideration for biomarker discovery thus lies in the selection of the appropriate training and testing datasets. Biobank recruitment and related large-scale omics data generation still favours European samples [47,48], precluding widespread translatability of the resulting biomarkers across diverse populations and generating bias. Approaches to address this have been previously reviewed [49]. Basic characteristics like age and sex have to be taken into account and, where indicated, models should be adjusted for these. In certain cases, it may be appropriate to train distinct models for males and females when biological evidence suggests the presence of differing underlying mechanisms. Importantly, demographic and socioeconomic characteristics are often not reported and thus ignored [50], despite their extensive influence on health outcomes (e.g. social deprivation index [51]). Furthermore, cohort-to-cohort differences can limit generalisability, which needs to be considered when integrating data from biobanks and real-world evidence. This may be due to different factors, including unaccounted biological variation, differences in technology (e.g. batch effects in lab-based assays) or sample/participant selection.

### AI algorithms

**Figure 2:**
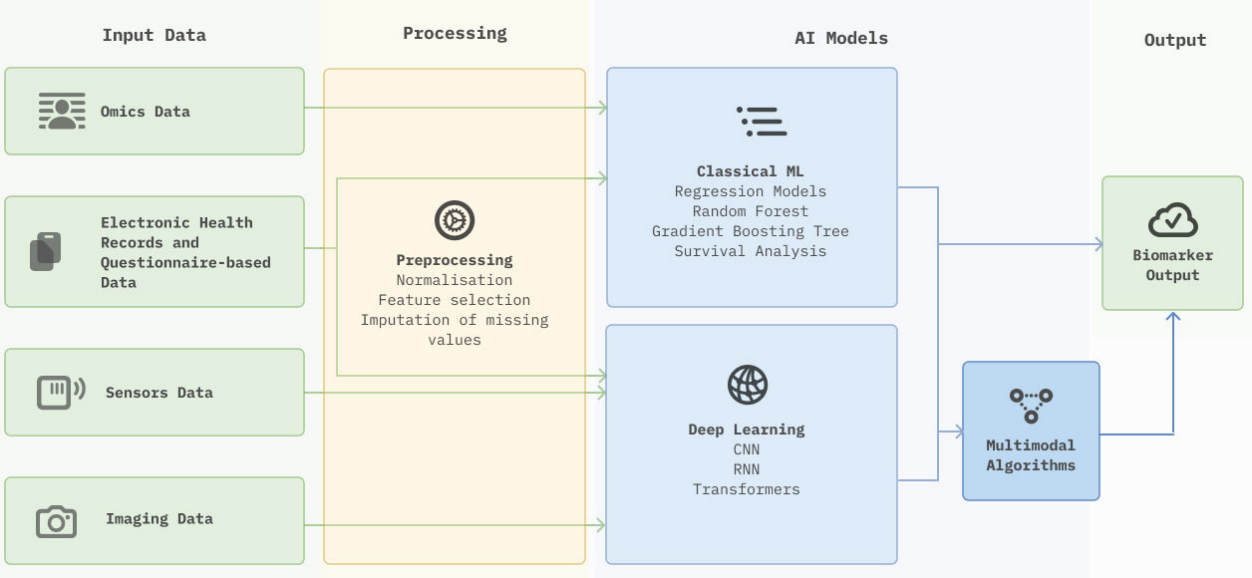
Flow diagram for AI-powered biomarker discovery. This figure illustrates which datatypes are typically processed using different AI approaches: classical machine learning (ML) and deep learning (DL) algorithms. CNN, convolutional neural network; RNN, recurrent neural network.

AI methodologies for biomarker discovery can be broadly categorised into two primary approaches: classical machine learning (ML) and deep learning (DL) (Figure 2). If multiple datatype modalities need to be considered, a multimodal infrastructure is required to integrate them, which usually leverages meta-learner algorithms trained on the output of other models that process different datatypes [52]. Selecting a suitable AI approach becomes a critical decision that affects the success and reliability of biomarker discovery, and it depends on different factors, such as the data available, the biomarker type and project constraints (like funding, know-how and infrastructure available).

Classical ML includes algorithms that are generally more interpretable, require well-crafted features and make predictions based on structured data, such as omics data, traditional blood and/or urine-based biomarkers (such as HbA1c or cholesterol), structured health records and questionnaire-based data. Some of the most popular classical ML algorithms include:

- Regression models. Elastic net regression models have been particularly useful in biomarker discovery, since they are able to deal with highly dimensional and redundant datatypes, such as those found when using epigenetic (DNA methylation) data [19]. The strength of regression models comes from their efficiency and interpretability. However, regression models struggle to capture more complex non-linear patterns [53].
- Random forest (RF) models belong to the ensemble learning category. These models use many decision trees in parallel, each trained on a subset of the data. RF models can capture non-linear patterns and process high-dimensional data with less propensity to overfitting. For instance, an RF classifier can be trained on 3.5 million genetic variants to predict Mendelian genetic disorders with more accuracy than existing methods [54]. RF models are more computationally expensive, and they require additional work for interpretability (feature importance).
- Gradient boosting tree models represent another ensemble method, with the difference that these models have the trees trained in sequence, allowing each tree to learn from the errors of the previous one. Thus, such models are effective at capturing how features interact with each other and can be used as the meta-learner for multi-modal solutions [55]. For instance, the multi-omics biomarker discovery framework MILTON uses an established and highly efficient implementation of gradient boosting (XGBoost) within a bespoke ensemble framework to build prediction models across 3000 diseases [46]. Like RF, model interpretability is possible by leveraging tree-based feature importance algorithms (e.g. split gain importance) [56] or more complex ones such as SHAP (SHapley Additive exPlanations) [57], which can be used to identify the relevant biomarkers for the outcome [58].

Survival analysis models predict the time until an event occurs, such as time-to-disease or death (mortality). Survival analysis models are efficient, lightweight and interpretable algorithms, making them favourable for building risk/susceptibility or prognostic biomarkers from omics data. The Cox proportional hazards regression model is popular and widely used to study the associations between different biomarkers and disease outcomes [19,59].

On the other hand, DL algorithms use large artificial neural network architectures to model and interpret complex data, which can be large, unstructured and noisy [1], such as unstructured temporal health data, clinical notes, sensor or imaging data. Some of the most popular DL algorithms include:

- Convolutional neural networks (CNNs) are DL models that can process multi-dimensional data structures, such as two-dimensional images (height × width) with multiple channels (like red, green and blue) or three-dimensional data (such as volumetric medical imaging). They allow automatic identification of spatial hierarchies of features (unlike in the case of classical ML, which normally requires more feature engineering). These models are widely used to train biomarkers from imaging data, for example, for the early prediction of Alzheimer’s disease (AD) from hippocampal MRI data [60]. The disadvantages of CNNs are that they primarily capture local dependencies within the data, they are computationally expensive, difficult to interpret and require large amounts of labelled training data.
- Recurrent neural networks (RNNs) are models used for processing sequential data. These models preserve a memory of inputs, which allows them to capture the temporal dependencies of the data (such as longitudinal EHR or sensor data). For example, RNNs have been leveraged to predict disease progression in the context of neurodegenerative disease [61]. However, a significant challenge for these models is their limited memory span, which makes it challenging for them to effectively utilise information from long-term dependencies in sequences.
- Transformers are models that use self-attention mechanisms to capture distant dependencies between data. LLMs like GPT (generative pre-trained transformers) [62] and BERT (bidirectional encoder representations from transformers) [63] are part of the transformer class and have recently increased in popularity. Transformer architectures have been used to train transcriptomic and epigenomic models that are able to both predict biological age and identify potential drug targets for age-related diseases [64]. One interesting recent application of transformers in the digital pathology domain has been in the development of foundation models. These foundation models are typically created using self-supervised learning techniques from extremely large cohorts of pathology images and have been shown to be the potential basis of state-of-the-art biomarker detection algorithms [65]. Furthermore, since transformers can use unsupervised learning, another application is processing unstructured textual data to construct a base knowledge of the context of information. For instance, data from PubMed, PMC and Wikipedia were processed to identify relevant information about rare diseases to aid their diagnosis and treatments [66]. Transformers face limitations such as high demands for large datasets and computational resources for training, along with ethical and privacy challenges.

Other AI algorithms frequently used to integrate multi-omics data include joint dimensionality reduction methods [67–69], generative adversarial networks (GANs) [20], graph neural networks [70,71] or graph transformer networks [72].

## Examples of AI-powered biomarker discovery

While we have tried to cover many of the commonly used AI algorithms in biomarker discovery, the literature on the topic is immense, and a systematic analysis is beyond the scope of this review. Some recent examples of the innovative application of AI in biomarker research are highlighted in Table 2, which includes different data modalities, therapeutic areas and AI tools.

**Table 2:** Examples of innovative AI-based biomarkers in various therapeutic areas and the toolkit used for their discovery.

Biomarker or framework name	Therapeutic area(s)	Datatype(s)	Biomarker categories	AI algorithm(s)	Regulatory status	Highlights	References
PRSmix	Many therapeutic areas	Genomics (germline)	Risk	Elastic net	Research use only	Improve accuracy of polygenic risk scores (PRS) across complex diseases and populations by aggregating existing PRS using ML.	[73]
AI-MARRVEL (AIM)	Mendelian genetic disorders	Genomics (germline)	Diagnostic	RF classifier	Research use only	Better and faster diagnosis of genetic Mendelian disorders by training a classifier on more than 3.5 million variants from thousands of diagnostic cases.	[54]
EpiSign	Mendelian genetic disorders	Epigenomics (bulk DNA methylation)	Diagnostic	Support vector machine (SVM)	Laboratory developed test	Identifies episignatures (methylation patterns associated with Mendelian disorders) from peripheral blood samples. It leverages an SVM to analyse DNA methylation and generate a methylation variant pathogenicity (MVP) score to classify patients into 43 episignature groups.	[74]
CytoTRACE2	Oncology	Transcriptomics (single-cell RNA-seq)	Prognostic, predictive	Gene set binary network (GSBN)	Research use only	Tool to analyse and interpret RNA-seq data using a novel interpretable deep learning framework (GSBN). It can be used to find gene signatures predictive of disease or response to drugs such as immune checkpoint inhibitors and chemotherapy.	[75]
ProteinScores	Age-related diseases and mortality	Proteomics	Risk	Cox proportional hazards regression and elastic net	Research use only	By integrating multiple protein markers, it is possible to build biomarkers that predict disease onset (ten year risk) better than using more traditional markers (such as age, sex and a comprehensive set of 24 lifestyle factors, clinically relevant biomarkers and physical measures); including for different age-related diseases (type 2 diabetes, chronic obstructive pulmonary disease, Alzheimer’s dementia, ischaemic heart disease, Parkinson’s disease) and mortality (death).	[76]
CSF-based AD biomarkers	Alzheimer's disease (AD)	Proteomics	Risk, diagnostic, prognostic, monitoring	Subtype and Stage Inference (SuStaIn) algorithm	Research use only	Establish a model of AD progression using cerebrospinal fluid (CSF) biomarkers and the novel Subtype and Stage Inference (SuStaIn) algorithm. This could become a cost-effective tool that complements/eliminates the need for PET scanning.	[77]
Nightingale Health plasma-based metabolic biomarkers	Many therapeutic areas	Metabolomics	Risk	Cox proportional hazards regression	Conformité Européenne (CE)-marked	Create a large-scale metabolomics biomarker-disease association atlas across >700 diseases and >100k individuals and identify signatures for disease risk.	[59]
Gut Microbiome Wellness Index 2 (GMWI2)	Wellness	Microbiome (genomics)	Diagnostic, monitoring	LASSO-penalised logistic regression	Research use only	Developing a disease-agnostic universal biomarker of gut health which can distinguish between healthy and unhealthy individuals. It was trained using 8069 metagenomic samples from 54 studies and 26 countries.	[78]
Prothrombin Time International Normalised Ratio (PT INR)	Cardiovascular disease	EHR	Predictive, monitoring	Dense neural networks and recurrent neural networks (RNNs)	Research use only	Deep learning algorithms are used to predict patient response to warfarin, a common anticoagulant which shows high response variability among individuals, thus enabling more optimised dose adjustments.	[79]
Brainomix 360 e-ASPECTS	Stroke	Imaging (CT)	Diagnostic	Deep learning algorithms	FDA 510(k) and CE-marked	It uses DL algorithms to analyse CT scans and quantify ischaemic damage for acute ischaemic stroke patients. It is able to identify subtle changes which may be missed by human readers and therefore improve decisions on treatment.	[80]
MSIntuit	Colorectal cancer	Imaging (digital pathology)	Diagnostic	Convolutional neural networks (CNNs)	CE-marked	Pre-screening tool that identifies microsatellite instability (MSI) status of colorectal cancer patients from haematoxylin-eosin (H&E) stained slides. By ruling out ~50% of non-MSI cases, it is a cost-effective way to alleviate MSI testing burden in clinical practice.	[81]
Quantitative continuous scoring	Lung cancer	Imaging (digital pathology)	Predictive	Deep learning algorithms	Research use only	AI-based immunohistochemical imaging biomarker for scoring TROP2 expression in non-small cell lung cancer (NSCLC) and predicting response to a TROP2-directed antibody–drug conjugate.	[82]
Stratipath breast	Breast cancer	Imaging (digital pathology)	Prognostic	DL algorithms	CE-marked	Prognostic risk stratification of breast cancer patients from H&E-stained tissue sample images. This is particularly relevant for the intermediate-risk ER+/HER2− patients, since improved prognosis can provide guidance relating to treatment decisions for adjuvant chemotherapy, and thus reduce over- and undertreatment of patients.	[83]
Virchow2	Oncology	Imaging (digital pathology)	Diagnostic, prognostic, predictive	Transformer-based foundational model	Research use only	A self-supervised learning foundational model trained on 3.1 million histopathology whole slide images, with diverse tissues, originating institutions and stains. It can perform many tasks in digital pathology.	[65]
Alzheimer's Disease progression model	Alzheimer's disease (AD)	Imaging (MRI)	Risk, prognostic	Convolutional neural networks (CNNs), LASSO-regularised Cox proportional hazards regression	Research use only	Building time-to-event models from MRI features to predict progression of mild cognitive impairment (MCI) to Alzheimer's disease (AD), which can help to stratify risk of MCI patients.	[60]
Apple Watch AFib history	Atrial fibrillation (AF)	Wearables (smartwatch)	Diagnostic	DL algorithms	FDA 510(k)	Using the photoplethysmography sensor which measures changes in blood flow, it is possible to detect irregular pulses and atrial fibrillation. This demonstrates a real-world application of wearable technology for health continuous monitoring.	[84]
MILTON	Many therapeutic areas	Multi-omics (genomics, proteomics, EHR)	Risk, diagnostic	Gradient boosting (XGBoost) within a bespoke ensemble framework	Research use only	This ensemble machine-learning framework leverages multi-omics data to predict >3000 diseases, outperforming available polygenic risk scores and augmenting genetic association analyses.	[46]
Duet evoC	Oncology	Multi-omics (genomics, epigenomics)	Diagnostic, monitoring	LASSO-regularised logistic regression	Research use only	By combining measurements of 5mC and 5hmC in cfDNA from liquid biopsies, it enhances diagnostic accuracy in colorectal cancer detection (AUC = 0.95) compared with traditional approaches that conflate these markers (modified C, AUC = 0.66). The assay can also detect genetics if used at higher sequencing depth. Together, this method can improve the sensitivity of liquid biopsy tests for early cancer detection.	[85]
Precious1GPT	Ageing and age-related diseases	Multi-omics (transcriptomics, epigenomics)	Risk	TabTransformer	Research use only	This model predicts biological age using RNA-seq and DNA methylation, and it also identifies potential therapeutic targets for age-related diseases.	[64]
InflammAge	Systemic chronic inflammation and ageing	Multi-omics (epigenomics, proteomics)	Risk	Elastic net	Laboratory developed test	It leverages a framework that maps blood proteomics to saliva DNA methylation data. This can help to build accessible biomarkers for preventative healthcare, including those required to quantify hallmarks of ageing, such as systemic chronic inflammation.	[19]
Gene-SGAN	Neurological disease	Multi-modal (genomics, MRI)	Risk, diagnostic	Deep weakly-supervised clustering method based on generative adversarial networks (GAN) and variational inference (VI)	Research use only	Multi-modal integration of neuroimaging phenotypes and genetic variants, which allows identification of disease subtypes and associated endophenotypic signatures.	[20]
High-grade serous ovarian cancer (HGSOC) prognostic model	Ovarian cancer	Multi-modal (genomics, transcriptomics, proteomics, digital pathology)	Prognostic	RF classifier	Research use only	Multi-modal data (histopathological image features, genomics, transcriptomics and proteomics) are used to predict survival for ovarian cancer patients (five year AUC = 0.911) and stratify them into high- and low-risk groups with high accuracy (HR = 18.23).	[86]
ArteraAI	Prostate cancer	Multi-modal (EHRs, digital pathology)	Predictive	Convolutional neural networks (CNNs) and multimodal deep learning architecture	Laboratory developed test	By combining digital pathology images from pretreatment prostate tissue and clinical data, the algorithm is able to predict which prostate cancer patients will benefit from androgen deprivation therapy, therefore avoiding unnecessary toxicity and side effects.	[87]

ER+/HER2−, oestrogen receptor positive/ human epidermal growth factor receptor 2 negative.

While this table is not all-inclusive, several key trends in AI-powered biomarker discovery can be observed. In general, single-omic and multi-omics biomarkers (such as ProteinScores, EpiSign, InflammAge, Nightingale plasma-based biomarkers, Duet evoC, GMWI2 or MILTON) seem to leverage more classical ML algorithms, such as LASSO, elastic net regression or tree-based models [19,46,59,74,76,78,85]. When it comes to imaging or multi-modal biomarkers, DL approaches are more suited due to the complexity of the data. For example, Brainomix uses DL algorithms to analyse CT scans of ischaemic stroke patients (which are able to detect subtle changes that may be missed by clinical experts) [80,88]. DL is also widely leveraged for tissue-based digital pathology biomarkers for cancer [89,90]. These biomarkers may be alternatives to existing ones such as microsatellite instability (MSI) or, more interestingly, be novel imaging biomarkers which are not possible using traditional pathological tests [82,87].

It can be seen that a great number of published studies generate susceptibility/risk and diagnostic biomarkers, which have applications in preventative medicine and early disease detection. This is particularly important in areas such as oncology, where early detection is crucial for prognosis and patient survival [87]. Neurology and cardiology biomarkers are other hot areas for the use of AI tools (such as cerebrospinal fluid-based AD biomarkers or prediction of AD progression) as they can aid clinicians in better understanding disease risk, progression and improve decisions on treatment, as well as dosage selection [60,76,77,79,80,84,88]. Biomarkers for wellness and health monitoring are also becoming a focal point, such as gut health monitoring from microbiome data [78] and quantifying ageing and its hallmarks using epigenetic data [19]. Continuous monitoring and real-time tracking are also gaining traction through the power of AI to derive insights from longitudinal data, such as from wearables like smartwatches [84]. This demonstrates a shift in reactive healthcare to a more proactive preventative approach, brought on by the use of AI, which will reduce disease burden and improve population health.

AI-powered biomarker discovery is also changing the drug discovery process. This includes applications to improve novel target identification and design biomarker-informed patient recruitment strategies for clinical trials, which can improve trial success rate and deliver on the promise of precision medicine. Specifically, recent AI-based models [46] help us better identify misdiagnosed and/or undiagnosed cases, leading to augmented case-control cohorts and thus higher statistical power to detect strong genetic signals (previously undetected in baseline association studies). These signals can not only elucidate novel gene targets for a disease area but also help us triage among an existing pool of candidates, supported by additional types of evidence. Other AI frameworks that leverage multi-omics data for novel target identification have also been reported, such as PandaOmics, which was used to identify potential therapeutic targets for amyotrophic lateral sclerosis [91]. Moreover, biomarker-informed patient recruitment is particularly critical in prevention trials, especially in oncology, as it enables the selection of patients with homogeneous disease profiles. This approach enhances the likelihood of identifying populations that are more responsive to specific treatments, thereby maximising the drug’s efficacy potential [92].

Unfortunately, population bias is still commonplace, with most biomarkers being trained in European populations and with limited data from diverse ethnic groups [59,78]. Nevertheless, there is an increased focus on generalisability and external validation of biomarkers in more diverse cohorts. This is evidenced in studies such as GMWI2, which has been validated across 54 studies in 26 countries [78], or in recent efforts using DL approaches to improve polygenic risk scores across different ancestries [93].

## The challenges to go from biomarker discovery to the clinic

AI has notably contributed towards progress in biomarker discovery; yet significant challenges remain to translate these biomarkers to the clinic. High-level calculations show that only between 1% and 2% of all the biomarkers published in the literature actually achieve clinical adoption by healthcare providers and patients [94,95]. Furthermore, it has been estimated that in order to develop and bring to market a typical in vitro diagnostic (e.g. those that will require FDA review), it can take between 20 and 100 USD million and 3–7 years [96], normally followed by a long period before widespread adoption in clinical guidelines.

**Figure 3:**
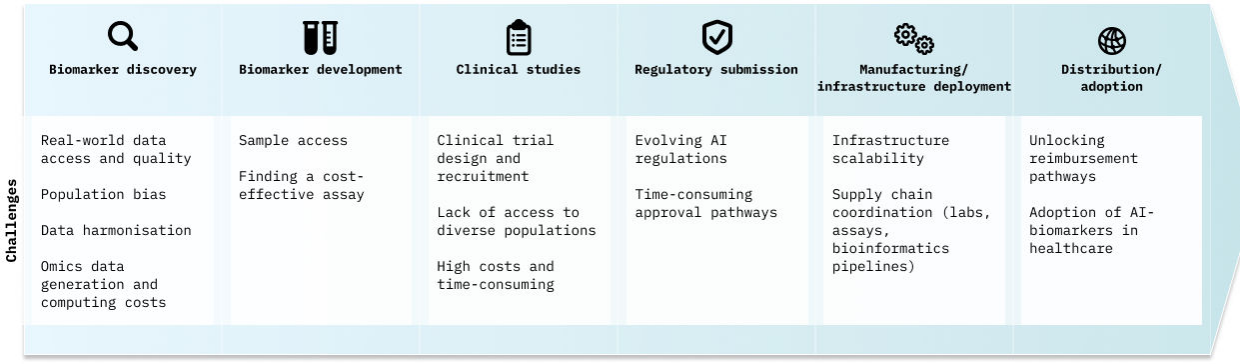
Key challenges that need to be considered to bring a biomarker from its early research stages to wide clinical adoption.

There are many challenges that need to be considered and addressed in order to see the fruits of AI in biomarker discovery having an impact on the real world (Figure 3). Challenges that affect diagnostics more broadly include cumbersome and expensive clinical trials (and access to samples), meeting new regulatory requirements or unlocking reimbursement pathways [97].

Importantly, there are certain challenges that particularly affect those biomarkers that may be discovered using AI. For example, accessing real-world data and real-world evidence from different populations (beyond biobank data, such as from hospitals) is paramount to avoid bias during model training, but this data is still heavily siloed and lacks harmonisation and interoperability [98]. In order to solve this problem, different global federated real-world data platforms are being developed, which can give access to large amounts of diverse and standardised data while protecting patient privacy and ensuring ethical use [99]. Furthermore, open-source common data models, such as OMOP (Observational Medical Outcomes Partnership), are paving the way towards data standardisation [100].

Additionally, multi-omics technologies are generally expensive, so it is important to consider the trade-off between accuracy and cost for a specific use case and disease during biomarker discovery, so that a viable health economics model can be proposed at the end. For instance, in the multi-omics biomarker discovery framework MILTON, researchers were able to demonstrate that adding a small number of proteomics biomarkers to a set of traditional clinical biomarkers is sufficient to significantly improve model performance in the prediction of specific diseases [46].

Infrastructure can become a bottleneck as well. Computational costs to train large DL models (particularly when there is a need to access GPUs) are significant, which is increasing the research gap between academia and industry, with fewer academics training models from scratch [101]. Furthermore, the diagnostics infrastructure becomes critical when different omics layers are combined in a single biomarker. This may require orchestrating a complex supply chain of different laboratories, assays and bioinformatic pipelines to be able to offer the final diagnostic at scale.

Despite these challenges, it is worth noting that the number of biomarkers with AI is increasing over time. When focusing on AI/ML-enabled medical devices, there are ~950 examples that have already passed FDA review [102], showing a bright future ahead for AI-powered biomarkers.

## The future of AI-powered biomarker discovery

AI has become a cornerstone of biomarker discovery, leading to the development of AI-powered biomarkers. In the coming years, we expect AI to also accelerate scientific discovery by enabling new forms of scientific collaboration and automation. One promising concept is that of a ‘Virtual Lab’. This is a system composed of multiple AI agents (powered by LLMs), each taking on the role of a virtual scientist (e.g. principal investigator, immunologist, computational biologist or machine learning specialist), and working together in a co-ordinated fashion to solve complex biomedical problems. For instance, a recent study demonstrated how such a Virtual Lab could design and implement a computational pipeline for nanobody development for new SARS-CoV-2 variants. This involved deciding on a strategy to modify existing nanobodies (rather than designing them *de novo*), generating candidate sequences, modelling nanobody-antigen structures and evaluating binding affinity *in silico*, all orchestrated by AI agents. The nanobody candidates proposed by the AI system were subsequently validated in wet-lab experiments and shown to bind emerging SARS-CoV-2 variants [103]. Therefore, it is not unthinkable to imagine a near future where multi-modal biomarker discovery is highly automated by AI agents. They could help to find the best biomarkers available in the literature for a given therapeutic area, suggest biomarker hypotheses (e.g. the best datatype for a particular application), generate code to train and test biomarker models faster than existing methods or guide doctors to accomplish better diagnoses by integrating all the available biological data and models for a given patient. This will require increased collaboration between experts from different fields, such as biologists, computational biologists, AI/ML engineers and doctors.

Clinicians are used to the concept of multi-modal diagnosis (e.g. when they review MRI results in the context of medical history). With the broader use of AI technologies to assist and automate this process, it will be critical that AI-powered biomarkers are interpretable and explainable, so they are adopted in clinical practice [104]. Advances in continuous biomarker monitoring through new wearable and sensor technology will lead to at-home real-time health monitoring, adding another dimension to quantify health and disease [105]. Importantly, these technologies need to be democratised and made accessible to all populations, so that the health equity gap and health inequalities are reduced [106]. AI has truly the potential to move biomarker discovery from the hardware layer (developing new ‘wet-lab’ assays) to the software layer (new insights from existing data and assays), therefore accelerating innovation cycles and the path towards precision medicine.

## Summary bullet points

- Artificial intelligence (AI) is driving innovation in healthcare research and biomarker discovery owing to its ability to derive insights from complex high-dimensional datasets and integrate multi-modal datatypes (such as omics, electronic health records, imaging or sensor and wearable data).
- To ensure that AI-powered biomarkers are generalised and unbiased, large datasets from diverse populations are required, such as biobanks, longitudinal studies, real-world data and data from clinical trials.
- Classical machine learning models (like elastic net regression and random forests) are more commonly utilised for single-omics and multi-omics biomarkers, whereas deep learning models (like convolutional neural networks or transformers) are important in handling datatypes like imaging, large unstructured electronic health records or multi-modal data.
- Key challenges to translating AI-powered biomarkers to the clinic and driving adoption need to be addressed, including lack of population diversity, difficulties accessing harmonised data, costly and time-consuming clinical studies, evolving AI regulatory frameworks and absence of scalable diagnostic infrastructure.
- AI agents will likely automate and enhance many steps of the biomarker discovery process, accelerating the paradigm shift towards precision medicine.

## Open Access

This article has been published open access under our Subscribe to Open programme, made possible through the support of our subscribing institutions, learn more here: https://portlandpress.com/pages/open_access_options_and_prices#conditional

## Abbreviations

AD: Alzheimer’s disease
AF: atrial fibrillation
AI: artificial intelligence
AUC: Area Under the Curve
BERT: Bidirectional Encoder Representations from Transformers
BEST: Biomarkers, EndpointS, and other Tools
C: Cytosine
CE: Conformité Européenne
CE-marked: Conformité Européenne - marked
CNNs: convolutional neural networks
CSF: cerebrospinal fluid
CT: computed tomography
2D: Two Dimensional
3D: Three Dimensional
DL: deep learning
DNA: Deoxyribonucleic Acid
ECG: Electrocardiograms
EEG: Electroencephalography
EHRs: electronic health records
ER+/HER2−: oestrogen receptor positive/human epidermal growth factor receptor 2 negative
FDA-NIH: Food and Drug Administration - National Institutes of Health
GANs: generative adversarial networks
GMWI2: Gut Microbiome Wellness Index 2
GNNs: Graph Neural Networks
GPT: Generative Pre-trained Transformer
GPUs: Graphics Processing Unit
GSBN: Gene Set Binary Network
GTNs: Graph Transformer Networks
GenAI: Generative Artificial Intelligence
H&E: Haematoxylin-Eosin
H&E: haematoxylin-eosin
HGSOC: high-grade serous ovarian cancer
HR: Hazard Ratio
HbA1c: Glycated hemoglobin
LASSO: Least Absolute Shrinkage and Selection Operator
LLMs: large language models
MCI: mild cognitive impairment
ML: machine learning
MRI: magnetic resonance imaging
MSI: microsatellite instability
MVP: methylation variant pathogenicity
NSCLC: non-small cell lung cancer
OMOP: Observational Medical Outcomes Partnership
PET: positron emission tomography
PMC: PubMed Central
PRS: Polygenic Risk Score
PT INR: Prothrombin time international normalised ratio
RF: random forest
RGB: Reg, Green and Blue
RNA-Seq: Ribonucleic Acid Sequencing
RNNs: recurrent neural networks
SARS-CoV-2: Severe Acute Respiratory Syndrome Coronavirus 2
SHAP: SHapley Additive exPlanations
SVM: Support vector machine
SuStaIn: Subtype and Stage Inference Algorithm
TREs: Trusted Research Environments
UK: United Kingdom
USD: United States Dollar
VI: variational inference
cfDNA: Cell-Free Deoxyribonucleic Acid
5hmC: 5-hydroxymethylcytosine
5mC: 5-methylcytosine
